# A single-crystal diamond X-ray pixel detector with embedded graphitic electrodes

**DOI:** 10.1107/S160057752000140X

**Published:** 2020-03-31

**Authors:** C. Bloomer, M. E. Newton, G. Rehm, P. S. Salter

**Affiliations:** a University of Warwick, UK; b Diamond Light Source Ltd, UK; c University of Oxford, UK

**Keywords:** CVD diamond, diagnostic, pixel, detector

## Abstract

The first experimental results from a new transmissive diagnostic instrument for synchrotron X-ray beamlines are presented. The instrument utilizes a single-crystal chemical-vapour-deposition diamond plate as the detector material, with graphitic wires embedded within the bulk diamond acting as electrodes. The resulting instrument is an all-carbon transmissive X-ray imaging detector. Within the instrument’s transmissive aperture there is no surface metallization that could absorb X-rays, and no surface structures that could be damaged by exposure to synchrotron X-ray beams. The graphitic electrodes are fabricated *in situ* within the bulk diamond using a laser-writing technique. Two separate arrays of parallel graphitic wires are fabricated, running parallel to the diamond surface and perpendicular to each other, at two different depths within the diamond. One array of wires has a modulated bias voltage applied; the perpendicular array is a series of readout electrodes. X-rays passing through the detector generate charge carriers within the bulk diamond through photoionization, and these charge carriers travel to the nearest readout electrode under the influence of the modulated electrical bias. Each of the crossing points between perpendicular wires acts as an individual pixel. The simultaneous read-out of all pixels is achieved using a lock-in technique. The parallel wires within each array are separated by 50 µm, determining the pixel pitch. Readout is obtained at 100 Hz, and the resolution of the X-ray beam position measurement is 600 nm for a 180 µm size beam.

## Abstract 2

The first experimental results from a new type of transmissive synchrotron X-ray diagnostic instrument are presented. A chemical-vapour-deposition diamond plate with graphitic electrodes and a modulation lock-in signal acquisition technique are used to image the X-ray beam.

## 1. Introduction   

Synchrotron light sources provide intense X-ray beams for researchers in a wide range of fields. The X-ray beam position, beam size, and beam intensity should remain stable for the duration of a synchrotron experiment. The typical requirements are that the beam position must be stable to better than 10% of the beam size, and intensity must be stable to 0.1% of the normalized intensity (Decker, 2005 ▸). Increasingly, these X-ray beams are focused to sub-micrometre sizes (Ice *et al.*, 2011 ▸), and maintaining the position and size of the beam is of critical importance. Variations in the incident flux intensity or beam profile can severely reduce the quality of the X-ray data collected. To ensure that these beam stability challenges are met, it is crucial to be able to non-destructively monitor synchrotron X-ray beams.

Single-crystal chemical vapour deposition (scCVD) diamond offers excellent properties for synchrotron X-ray beam monitoring. Under the presence of ionizing radiation, electron–hole generation occurs within the bulk diamond. An electric field applied across the diamond causes these charge carriers to flow to electrodes applied to the surface of the material, and the resulting current is proportional to the ionizing radiation flux. The excellent thermal conductivity, low thermal expansion, high melting point and radiation hardness of diamond make it particularly well suited for use as a detector of intense radiation beams (Friedl, 1999 ▸). It has a wide band gap and high carrier mobility, resulting in very low ‘dark’ currents and fast response times (Isberg *et al.*, 2002 ▸; Pomorski, 2008 ▸). The number of charge carriers generated is directly proportional to the power of the radiation deposited in the diamond across many orders of magnitude (Bohon *et al.*, 2010 ▸). Finally, its low atomic number and resulting low X-ray absorption and beam scattering make it an excellent choice for a transmissive X-ray detector (Bergonzo *et al.*, 2006 ▸; Morse *et al.*, 2007 ▸).

The use of diamond as a radiation detector is not a new development. The discovery that ionizing radiation can be used to induce an electric current within diamond was first observed in natural gemstones in 1941 (Stetter, 1941 ▸). However, natural gemstones are highly variable in quality and prohibitively expensive, making them unsuitable for application as radiation detectors. In the modern era it is the commercial availability of high-quality scCVD diamond that has allowed for a new generation of monochromatic synchrotron X-ray diagnostics. This technique for transmissively monitoring the position and intensity of X-ray beams has become common at synchrotron light sources. Such diagnostic instruments are used for beamline commissioning and alignment, used in feedback loops to keep the X-ray beam focused on the experimental sample, and used to record variations in the beam position over time (Morse *et al.*, 2007 ▸; Bloomer & Rehm, 2017 ▸).

A diamond profile monitor is advantageous due to its lower X-ray absorption and excellent thermal properties compared with a fluorescent screen. The absorption of the incident beam from 100 µm of scCVD diamond is <5% of the incident beam for 10 keV photons, compared with >80% for an equivalent thickness of fluorescent material for beam imaging (Henke *et al.*, 1993 ▸).

**Figure 1:**
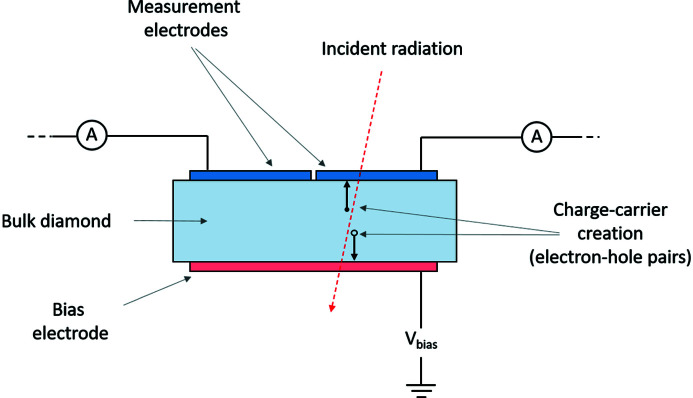
Schematic diagram of a position-sensitive diamond radiation detector. For clarity, the single ‘bias’ electrode is coloured red, and the ‘measurement’ electrodes are coloured blue.

A sketch of a position-sensitive radiation detector is given in Fig. 1 ▸. A single ‘bias’ electrode is located at the bottom of the device, covering the whole surface. Separate ‘measurement’ electrodes are applied to the top surface of the device. The generated charge carriers travel through the diamond under the influence of the electrical bias until they are collected by one of the electrodes. This current is measured by electrometers or other sensitive ammeters. A simple 2 × 2 arrangement of four electrodes is commonly used to provide position sensitivity in two dimensions (Morse *et al.*, 2007 ▸; Bloomer & Rehm, 2017 ▸), with the imbalance in the current readout from the different electrodes giving sensitivity to position of the incident beam. It has also been demonstrated that a series of strip-electrodes on the surface of a diamond plate can be used in order to measure the one-dimensional or two-dimensional (2D) beam profile (Shu *et al.*, 1999 ▸; Zhou *et al.*, 2015 ▸). This beam profile information is advantageous as it allows real-time monitoring of the beam shape, and can be used to align and optimize beamline optics.

The isolation gap between ‘measurement’ electrodes is generally as small as practical, typically between 2 µm and 20 µm. Smaller isolation gaps have been shown to improve the position resolution of the detectors, particularly for very small X-ray beam sizes (Griesmayer *et al.*, 2019 ▸). However, the electrical isolation between these neighbouring electrodes must be good, typically of the order GΩ, as electrical leakage between electrodes will modify the measured signal currents. This introduces uncertainties into the beam position measurement.

This very small isolating gap between neighbouring surface electrodes is found to be a source of detector failure (Bloomer *et al.*, 2018 ▸). Damage to the electrodes may occur during handling or installation, and dust or other debris may adhere to the metallization and short the isolating gap. Incident X-rays passing through an oxygenated atmosphere generate ozone which can degrade the metal electrodes themselves through oxidation (Bohon *et al.*, 2010 ▸; Zhou *et al.*, 2015 ▸). The diamond plate itself is resistant to these damage mechanisms, leaving the metallization as the weak point. A related issue is that of a thin film of conductive carbon forming on the surface of the device during exposure to beam, due to cracking of atmospheric hydrocarbon molecules by photoelectrons emitted from the surface of the detector. This is an understood phenomenon (Boller *et al.*, 1982 ▸), and the resulting conductive layer can reduce the electrical isolation between two neighbouring electrodes from ∼GΩ to a few 100 Ω.

It should be noted that, despite these problems, diamond X-ray diagnostic instruments are used on many synchrotron beamlines. Careful handling and installation procedures, and use of the detectors in vacuum, help to mitigate these issues. However, the development of more robust diagnostic detectors would be of significant importance.

In this paper, a pixelated, transmissive X-ray detector is presented, and we aim to meet the need for more robust instruments by removing the necessity for any surface metallization within the instrument’s transmissive aperture. There are no surface structures within the beam path that could be damaged by exposure to synchrotron X-ray beams. To achieve this, ultra-short laser pulses have been used to ‘write’ conductive graphitic electrodes within the bulk diamond. This technique was first developed to fabricate graphitic columns to form electrical contacts with buried semiconductor layers within CVD diamond (Walker *et al.*, 1997 ▸), and was later adapted for detector applications (Oh *et al.*, 2013 ▸; Caylar *et al.*, 2013 ▸).

## 2. Detector design   

The prototype detector presented in this paper is fabricated using an ultra-short pulse laser technique which enables the fabrication of graphitic wires following arbitrary 3D paths within diamond plates (Sun *et al.*, 2014 ▸). This technique makes it possible for the first time to laser fabricate a detector with embedded conductors running parallel to the front face of the diamond. The ability to write electrodes in this geometry within the bulk diamond overcomes several of the challenges that have been discussed above, as the electrodes can be ‘buried’ underneath the diamond surface. This provides protection from mechanical and chemical damage, and from conductive surface contaminants. The graphitic electrodes are more transmissive to X-rays than an equivalent thickness of aluminium, titanium, or other traditional electrode material (Henke *et al.*, 1993 ▸). Additionally, there is no danger of the electrode material introducing new absorption edges that may affect synchrotron experiments, as the *K*-edge in both graphite and diamond (carbon *sp* ^2^ and *sp* ^3^, respectively) only differ by a few eV (Hamon *et al.*, 2004 ▸).

**Figure 2:**
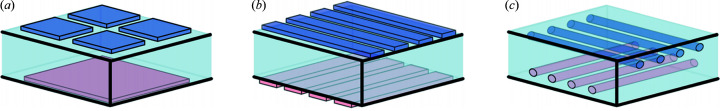
Sketch (not to scale) illustrating different detector electrode designs that are currently in use (*a*, *b*), and the electrode design presented in this paper (*c*).

Fig. 2 ▸ presents several electrode layouts that have been developed for X-ray diagnostic instruments. Fig. 2(*a*) ▸ shows a typical electrode layout in current use at synchrotrons for X-ray beam position monitoring applications, in a 2 × 2 arrangement. Fig. 2(*b*) ▸ shows a strip-electrode layout whereby two sets of orthogonal strip-electrodes are used, on opposite faces of the diamond plate. A bias voltage is applied to one ‘bias’ strip at a time, and the signal currents from each of the orthogonal ‘measurement’ strips on the opposite face are read, providing the measurement for one row of pixels. The bias voltage is rotated to the next strip, reading out the next row of pixels, and so on (Shu *et al.*, 1999 ▸; Zhou *et al.*, 2015 ▸). Fig. 2(*c*) ▸ depicts the detector discussed in this paper. It uses a set of laser-written graphitic wires as the strip-electrodes, buried under the surface of the diamond.

**Figure 3:**
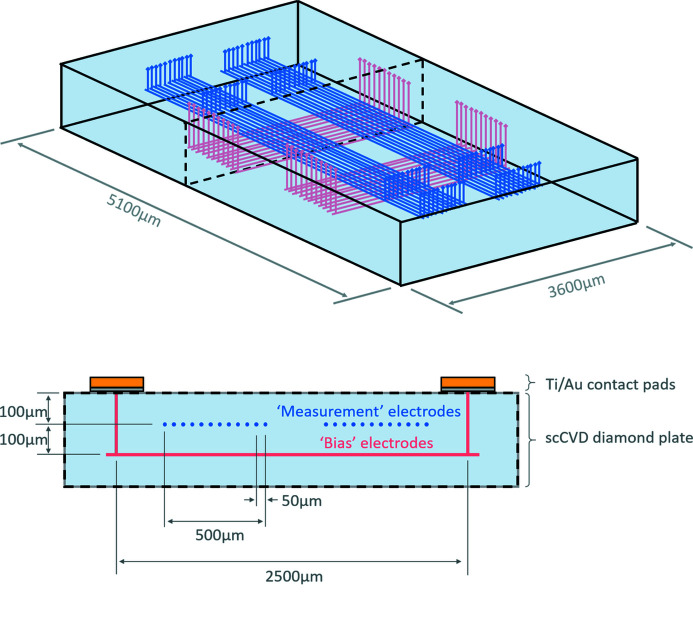
Top: sketch (not to scale) of the wire layout within the diamond plate. For clarity, the ‘bias’ electrodes are coloured red and the ‘measurement’ electrodes are coloured blue. A black dotted line indicates the region of the cross-section presented. Bottom: cross-section through the diamond plate. The location of the surface metallization at the edge of the detector is shown.

Fig. 3 ▸ shows the layout of the detector. The diamond is an optical grade scCVD plate, 5.1 mm × 3.6 mm in size and 600 µm thick. The measurement array is located at a depth of 100 µm within the material, and the bias array is located at a depth of 200 µm, providing an electrode array separation of 100 µm within the diamond. There is an in-plane spacing of 50 µm between each individual electrode within each array. Each array comprises of a group of 11 electrodes, a pattern which was repeated twice in order to provide redundancy in case of the graphitic tracks or the surface contact being incomplete or non-conductive. This presents a set of 22 bias electrodes and 22 measurement electrodes. With two sets of 11 bias electrodes and two sets of 11 measurement electrodes there are four potential 11 × 11-pixel detector regions, with a pixel pitch of 50 × 50 µm.

**Figure 4:**
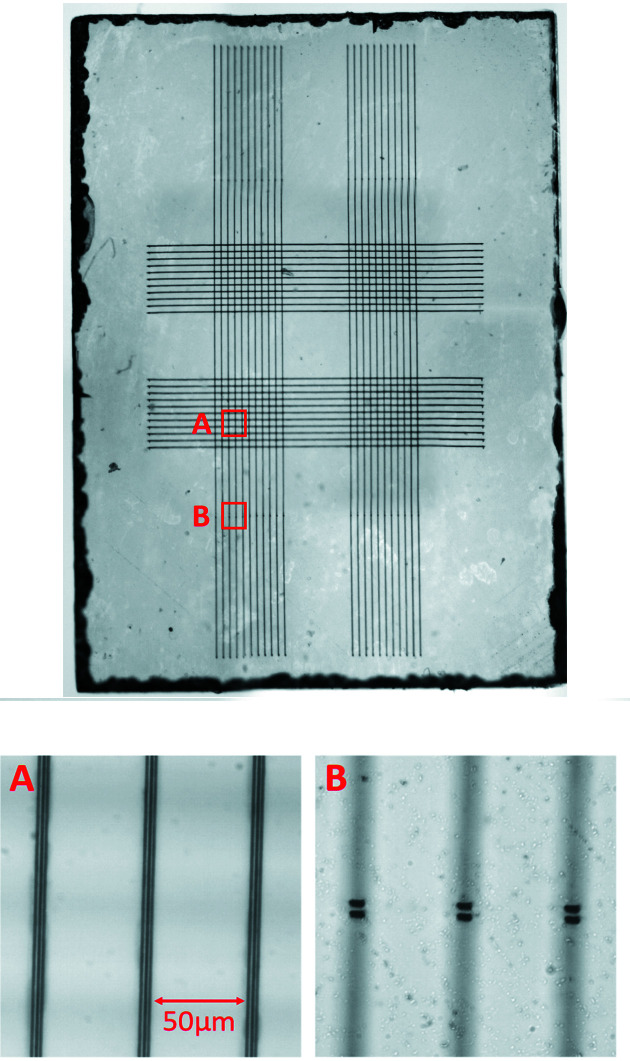

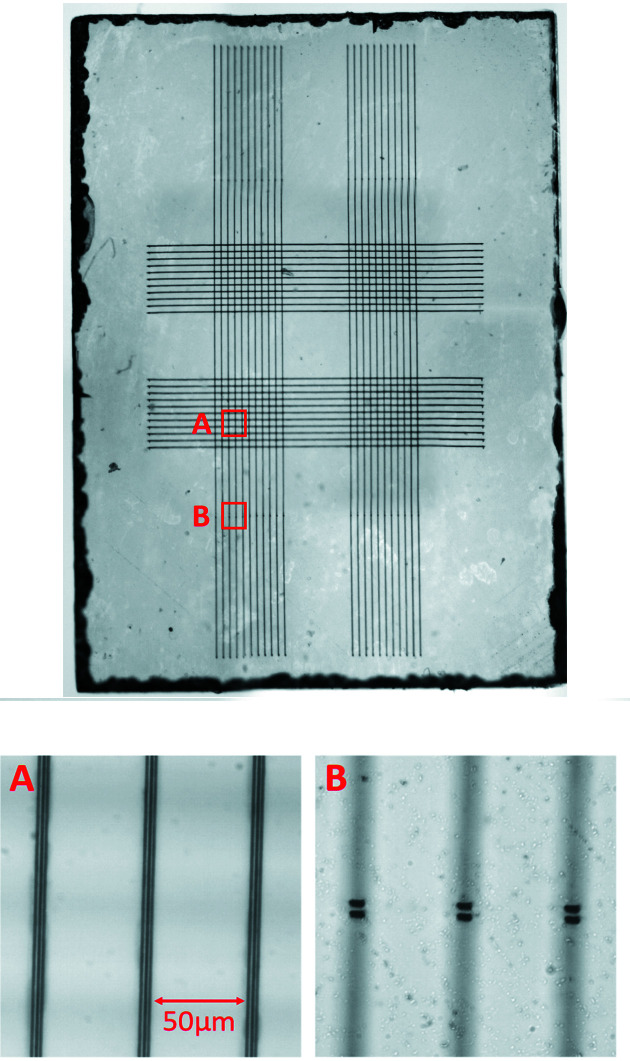
Top: composite microscope image of the diamond plate showing the fabricated graphitic wires. Bottom-left: magnified view of the area shown in the red square marked ‘A’. The upper electrodes can be seen in focus; the lower electrodes are out of the microscope focus. It can be seen that each electrode comprises three parallel tracks laterally spaced by 2 µm. Bottom-right: magnified view of the area shown in the red square marked ‘B’. The upwards ‘vias’ are visible in focus.

The laser processing was implemented using an amplified Ti:sapphire laser with a wavelength of 790 nm, pulse repetition rate of 1 kHz and pulse duration of 200 fs. The pulses were focused at high numerical aperture (NA = 1.4) inside the diamond using an oil-immersion objective lens. A liquid-crystal spatial light modulator was employed to compensate depth-dependent optical aberrations and ensure that the laser fabrication was the same at each depth (Sun *et al.*, 2014 ▸). Each in-plane electrode is fabricated using three individual graphitic tracks separated laterally by 2 µm. Each graphitic track has a width of approximately 1 µm. These can be seen in the microscope image of the tracks in Fig. 4 ▸. Each of these tracks was made with six passes of the diamond plate through the laser focus at a speed of 100 µm s^−1^ and a laser pulse energy of 50 nJ. To allow current to flow from the electrodes to the surface of the diamond, vertical graphitic ‘vias’ were subsequently written into the diamond using the same technique. As seen in Fig. 4 ▸, each of these vertical columns comprises two individual graphitic tracks, both of which connect to all three of the lateral tracks in each electrode. Multiple graphitic tracks are used for the electrodes and the columns to improve the conductivity of the wires. A total of four upwards ‘vias’ were added to each measurement electrode for use in future work exploring the conductivity of the graphitic tracks.

The total laser processing time required for this device was 150 minutes, and the process could be easily modified to increase the pixel count in a future detector. By using a different laser source with a higher pulse repetition rate, this time could easily be reduced by an order of magnitude, such that larger 100 × 100 pixel devices would be feasible.

A Ti–Au ohmic metal contact pad was applied to the diamond surface, where the upward ‘via’ reached the surface. Due to a problem with the application of the metallization, two out of the 44 total electrodes had incomplete surface pads and were unusable. This resulted in two 11 × 10-pixel usable detector regions, and two 11 × 9-pixel usable regions being available. The problems encountered during the metallization steps are understood to be primarily due to edge-beading, and will be addressed so that future detectors are not affected by this problem.

The diamond plate was fixed to a custom printed circuit board, and each surface pad on the diamond was wire bonded to a corresponding track on the board. The board had an aperture located underneath the diamond plate to allow transmission of the X-ray beam.

The overall 600 µm thickness of the diamond plate was dictated only by the availability of material. The relatively large 100 µm separation between the measurement and bias electrodes within this prototype detector was chosen to increase X-ray absorption and charge carrier generation. The 100 µm separation between the surface of the diamond plate and the upper electrode array was arbitrarily chosen, and for a future detector could be ∼5 µm.

Ideally, a transmissive detector would remain as thin as practical so as to minimize X-ray absorption. The successful results presented in this paper demonstrate that a thinner detector, fabricated from a ≤50 µm-thick diamond plate, would be feasible and would generate sufficient signal currents. Future work will aim to fabricate a thinner detector with a larger pixel count. The techniques involved are now established technologies and could be reliably scaled up to produce detectors with a larger pixel count, or to produce a small series of similar detectors.

## 3. Birefringence imaging of the diamond plate   

**Figure 5:**
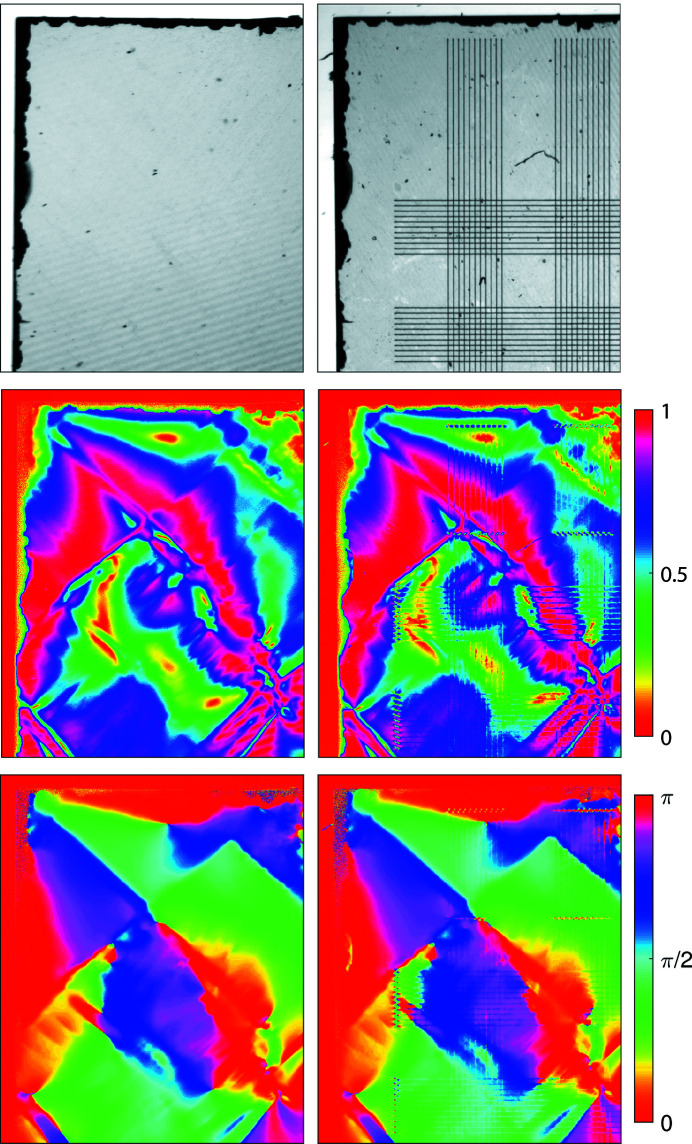
Birefringence imaging microscopy from the diamond plate before and after wire fabrication (left and right images, respectively). Top: transmission intensity maps. Middle: |sinδ| retardation maps (anisotropy). Bottom: orientation maps. Image width: 2.8 mm.

Birefringence imaging microscopy using a rotating polarizer can evaluate the cumulative lattice strain and orientation through a diamond sample (Glazer *et al.*, 1996 ▸). A birefringence imaging system (the Metripol Birefringence Imaging System, Oxford Cryosystems Ltd) was used to observe the effect of the written wires on the lattice. The results are shown in Fig. 5 ▸. These images show that some strain is introduced into the diamond by the fabricated wires, but that any strain induced is local to the wires themselves. It is small with respect to the existing strain found within the diamond plate, and with respect to that generated by the edge-chipping that occurred during the plate preparation. The laser-induced strain is most visible around the area of the upwards ‘vias’, due to the greater optical path length. This provides evidence that the laser-writing process does not substantially damage the single-crystal nature of the diamond itself in the unprocessed regions.

## 4. Electric field modelling   

Charge carriers generated within a diamond plate under the presence of an electric field are very quickly accelerated to their maximum drift velocities. For small electric field gradients and small distances, where the charge carriers do not reach their saturation velocities, this drift velocity is determined by the local electric field, **E**, and their mobility, μ. This results in a drift velocity that is directly proportional to the electric field, **v** = μ**E**. Carrier lifetime in scCVD diamond is reported in the literature to be in the range of a few 10 ns for optical grade material as used in this work, or up to ∼1 µs for electronic grade diamond plates (Isberg *et al.*, 2002 ▸; Pernegger *et al.*, 2005 ▸; Lohstroh *et al.*, 2007 ▸). These lifetimes are long enough that bias voltages of just a few 10 V (0.1 V µm^−1^) are sufficient for complete charge collection for the detector design presented in this paper.

**Figure 6:**
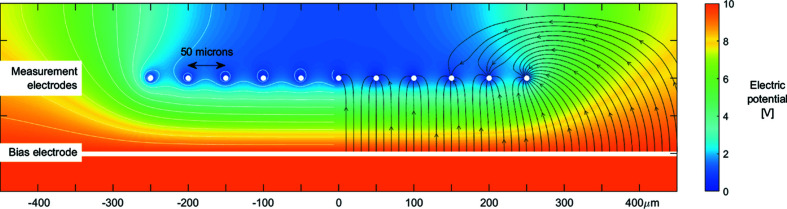
A simulation of the electric potential within the diamond plate. A static bias is applied to the bias electrode, and the measurement electrodes are grounded. The left half of the image depicts equipotential lines; the right half depicts field lines indicating the path of electrons travelling towards the measurement electrodes under the influence of the electric field.

Presented in Fig. 6 ▸ is the electric potential within the diamond cross-section highlighted in Fig. 3 ▸, computed using 2D finite element analysis (QuickField Professional, Tera Analysis Ltd). Applying a constant bias voltage to each of the bias electrodes within the detector will result in a nearly uniform electric potential within the diamond plate between the ‘bias’ and ‘measurement’ wires. Any charge carriers generated within this potential will follow the gradient until they reach one of the measurement electrodes. Electric field lines are also shown in Fig. 6 ▸, indicating the flow of charge carriers under the influence of the bias voltage.

## 5. Experimental results using a static bias   

To test the response of the detector to incident X-rays, it was installed in air at the sample point of the I18 beamline at Diamond Light Source (Mosselmans *et al.*, 2009 ▸). The beamline was configured to deliver a collimated flux of ∼1 × 10^11^ photons s^−1^ at 10 keV. The detector was mounted on an *XY* stepper motor stage. One of the two 11 × 10 pixel-detector regions (11 measurement electrodes and 10 bias electrodes) was chosen arbitrarily, and the detector aligned using the stepper motor so that the X-ray beam was transmitted through this pixel array.

**Figure 7:**
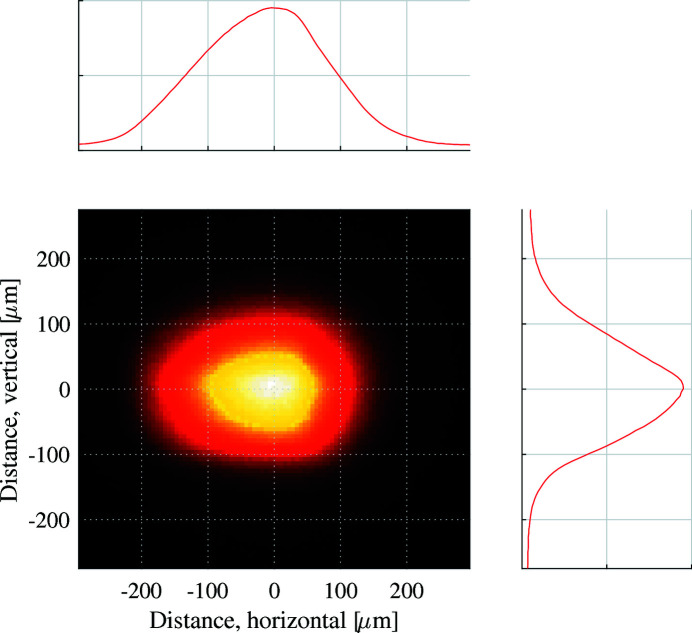
The sample-point X-ray beam size and shape recorded by the X-ray camera. The beam size is 240 µm horizontally and 180 µm vertically FWHM

An X-ray camera (a cerium-doped lutetium–aluminium–garnet, LuAG:Ce, fluorescent screen recorded with a CMOS sensor, effective pixel size of 5.9 µm) was installed directly behind the detector, 20 mm downstream of the diamond plate. The beam profile measured by the camera is presented in Fig. 7 ▸, and measured to be 240 µm horizontally full width at half-maximum (FWHM) and 180 µm vertically FWHM.

**Figure 8:**
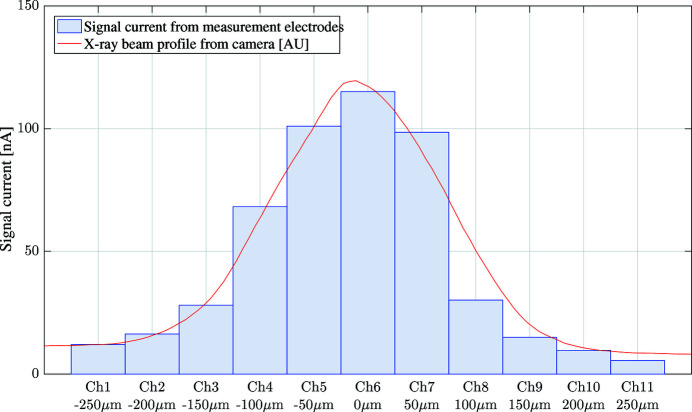
The photoionization currents reaching each of the 11 horizontal measurement electrodes embedded within the diamond plate are presented, providing the one-dimensional vertical X-ray beam profile. The vertical beam profile recorded by the X-ray camera downstream of the diamond is also shown on the plot.

Initially a static bias of +10 V was applied to all of the bias electrodes. In this configuration the centre of the pixel detector experiences a nearly homogeneous electric field gradient, as shown in Fig. 6 ▸. Generated charge carriers will flow along the field gradient to the nearest measurement electrode. With the X-ray beam striking the centre of the detector, a position of 0 µm as seen in Fig. 6 ▸, the signal currents collected on each measurement electrode was recorded by a low-impedance electrometer. The magnitude of the signal currents is directly proportional to the absorbed photon power, providing a one-dimensional profile of the X-ray beam. A comparison of the beam profile measured by the detector with that obtained by the X-ray camera is presented in Fig. 8 ▸. These two measurements of the X-ray beam profile show good agreement.

**Figure 9:**
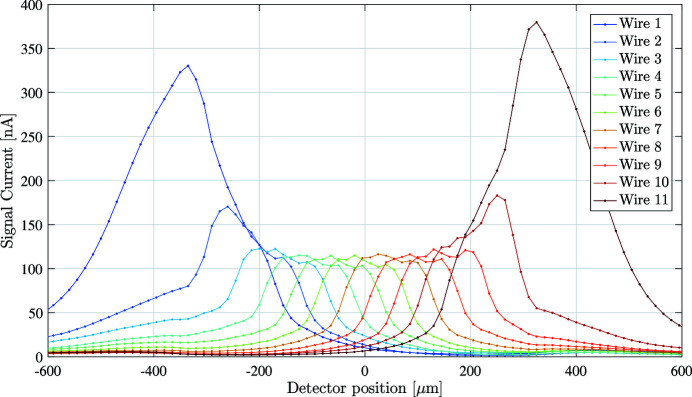
Using the stepper stage to vertically scan the detector through the X-ray beam to record the signal current reaching each measurement electrode at each step.

By driving the stepper stage, the beam can be scanned across the face of the pixel-detector. Presented in Fig. 9 ▸ are the signal currents measured during a 1200 µm vertical stepper motor scan across the face of the 500 µm-wide pixel-detector region.

As the detector is scanned through the beam, each measurement wire in turn collects charge generated by the absorbed X-rays. The figure shows that the outer wires, 1 and 11, collect a significantly greater amount of current. This is understandable, and is due to charge carriers generated outside of the pixel array flowing along the electric field gradient towards the outermost measurement wire, as illustrated in Fig. 6 ▸. The carrier lifetime dictates how far away from the pixel array charge carriers can be generated and still be collected. Future work will concentrate on mitigating this effect through the use of laser-written ‘guard rails’ to better confine the electric field within the diamond plate and to prevent charge carriers generated outside of the array from reaching the measurement electrodes.

## 6. Bias voltage scans   

The bias voltage influences the charge collection efficiency of the device. To determine the impact of the bias voltage, the X-ray beam was centred on one of the measurement electrodes, and the bias voltage on all of the bias electrodes was swept from −30 V to +30 V. (Higher voltages were not used during this charge collection experiment due to concern of damaging the detector.) This bias scan was then repeated for multiple measurement electrodes, carefully aligning the X-ray beam so that it was centred on each electrode in turn. As discussed in the previous section and illustrated in Fig. 9 ▸, the outermost measurement electrodes will accumulate charge carriers that are generated outside of the pixel array, potentially collecting more charge in total than the inner electrodes. Because of this, only the inner measurement electrodes (wires 3–9) were used for this experiment.

**Figure 10:**
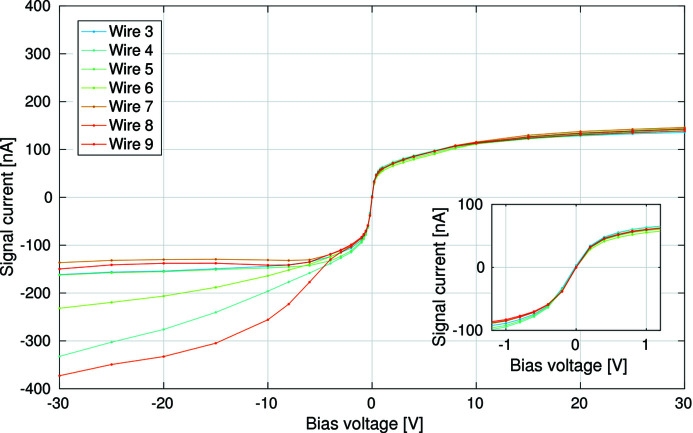
An *I*–*V* measurement, recording the current observed by each of the central measurement electrodes (3–9) when aligning the detector such that they intercept the beam centre. The voltage applied to the bias electrodes is swept from −30 V to +30 V. The inset shows the detail in the region −1 V to +1 V.

Fig. 10 ▸ presents the results of this bias sweep. A positive bias voltage results in very consistent results between measurement electrodes, with +20 V sufficient to collect ∼90% of the negative charge carriers (electrons). Based on these data, full charge collection is estimated to occur at 30–50 V. With a separation between the bias and measurement electrodes of 100 µm this represents a saturation voltage of 0.3–0.5 V µm^−1^. This measured saturation voltage is in line with previously published values (Pacilli *et al.*, 2014 ▸; Conte *et al.*, 2015 ▸).

While the response of our device to positive bias voltages is typical for this type of detector, the response to negative bias voltages is highly atypical. The flow of positive charge carriers (holes) to the measurement electrodes varies between measurement electrodes when a bias voltage of less than −3 V is applied. It is unclear as to why this is the case, and is under further investigation.

## 7. Experimental results applying a modulated bias   

**Figure 11:**
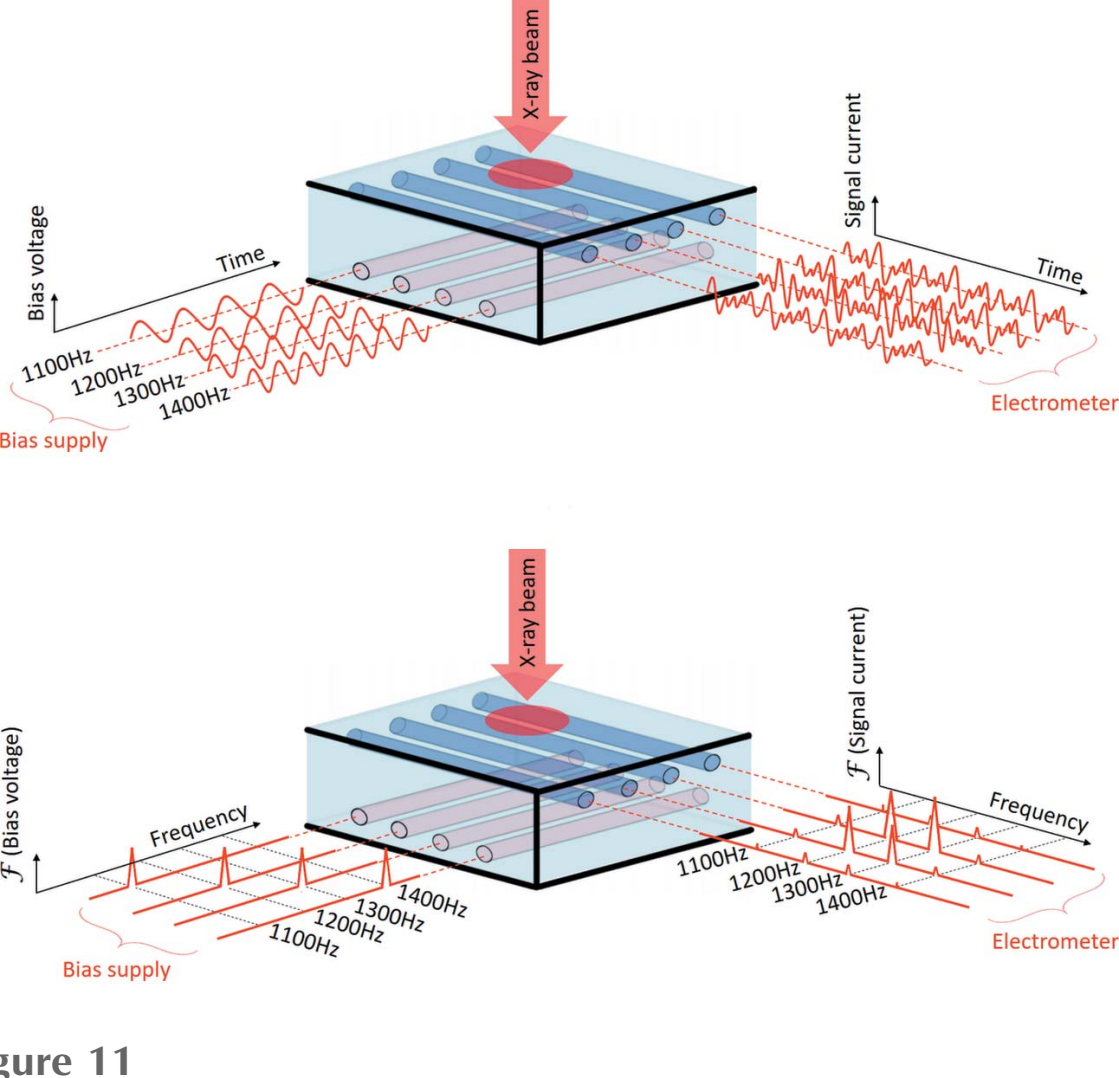
The readout scheme presented in this work utilizes a different modulation frequency applied to each bias electrode. The individual modulation frequencies are detectable at the measurement electrodes. Top: time-domain view of the modulation scheme. Bottom: frequency-domain picture, showing how the individual modulation frequencies measured by the electrometer build up a picture of the beam profile upon the detector.

The fabricated device is capable of obtaining two-dimensional images of the X-ray beam. It would be possible to apply the bias voltage to just one electrode at a time, record the signal currents, and then cycle the bias voltage to the next electrode in order to build up an image of the beam. This has previously been demonstrated in similar detectors (Shu *et al.*, 1999 ▸; Zhou *et al.*, 2015 ▸). However, this technique does not allow all pixels to be acquired simultaneously: only one ‘row’ of pixels may be read out at a time. It is limited by the speed at which bias switching can be driven, and having to ensure that the readout periods are synchronized with the switching. A more elegant solution is to modulate the bias voltages applied to each electrode with a known frequency, and to use Fourier analysis of the resulting signal currents to determine which bias electrode the charge carriers originated from. An illustration of this approach is presented in Fig. 11 ▸.

A multi-channel 16-bit digital-to-analogue converter (DAC) with 20 kHz update rate was used to supply each bias electrode independently with a modulated voltage. Due to the problem with the application of the surface metallization, 10 sequential bias electrodes were available for use out of a possible 11. The bias modulation frequencies chosen were 1.0 kHz, 1.1 kHz, 1.2 kHz,…, 1.9 kHz, and a modulation amplitude of 0.5 V around a DC level of 0 V was used. As can be seen in Fig. 10 ▸, a small modulation amplitude is desirable to keep the signal response linear with respect to bias voltage. Large modulation amplitudes result in non-linearities as the charge collection begins to saturate.

**Figure 12:**
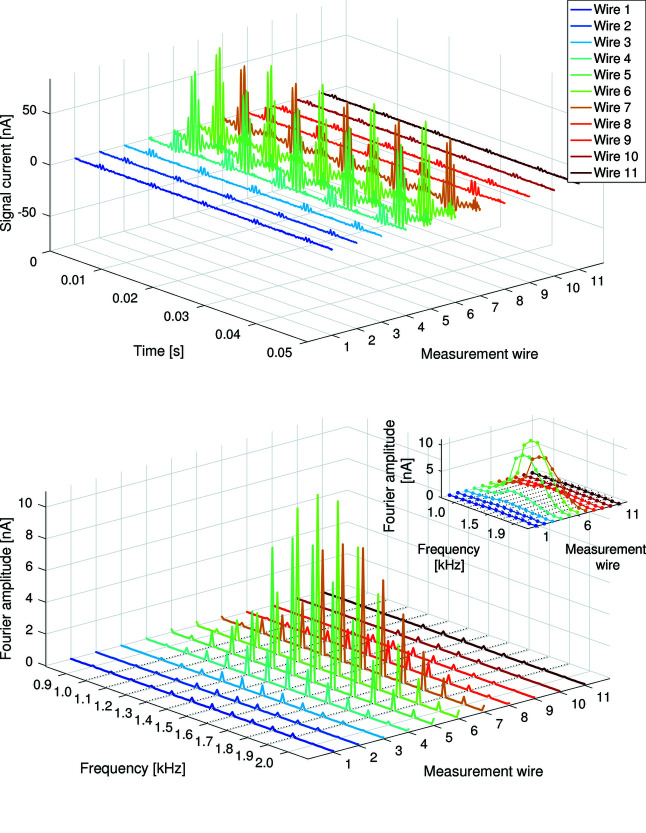
Top: 20 kHz signals acquired from 11 measurement electrodes whilst the X-ray beam is illuminating the detector. Bottom: Fourier transform of a 1 s-long acquisition at 20 kHz signals. Inset: Fourier transform of a 10 ms acquisition, such that each frequency bin of the discrete Fourier transform corresponds to one column of pixels on the detector.

**Figure 13:**
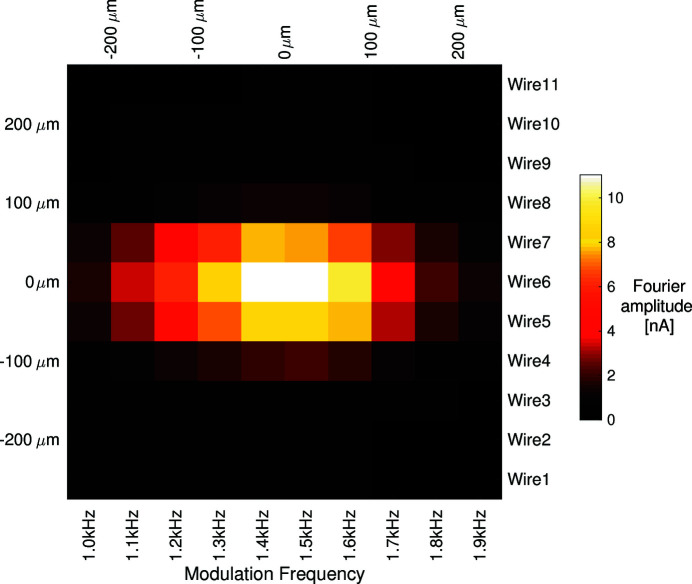
The reconstructed image of the X-ray beam footprint on the detector.

The resulting signal currents were acquired using low-impedance electrometers with a 5 kHz analogue low-pass filter. A 20 kHz analogue-to-digital converter (ADC) digitized the results. The measured signal currents and their Fourier transforms are presented in Fig. 12 ▸. Fig. 13 ▸ presents the image of the beam obtained from the amplitude of the modulation frequencies. Each of the measurement wires corresponds to one ‘row’ of pixels, and each of the modulation frequencies corresponds to one ‘column’ of pixels. The 50 µm electrode spacing determines the pixel pitch, and thus the exact size of the beam footprint upon the detector is obtained.

The choice of modulation frequencies used is not arbitrary: the range has been specifically chosen as it easily allows for short acquisition periods of just 10 ms, allowing 100 frames per second (FPS) acquisition of the images. With a 20 kHz sampling rate, a 10 ms acquisition will contain 200 samples. The discrete Fourier transform of these samples will result in each of the modulation frequencies occupying exactly one frequency bin, simplifying the image acquisition. The inset in Fig. 12 ▸ presents the Fourier transform from one 10 ms acquisition to illustrate this.

Once the image is acquired, a 2D Gaussian fitting routine is used to determine the location of the beam centre. In this way, the X-ray beam position and size can be measured at 100 Hz.

To determine the precision of the detector, the measured beam position was recorded as the instrument was stepped vertically through the X-ray beam using the stage. At each position 10 s of images were acquired at 100 FPS. For the smallest step sizes, the requested motion was sufficiently small that the stage resolution was insufficient to produce regularly spaced steps. The smallest requested step size was 3 µm; however, the stage positioning accuracy was only ±1 µm. Thus, at each step of the scan the actual beam position was recorded using the X-ray camera mounted on the motion stage, observing the X-ray beam transmitted through our detector.

**Figure 14:**
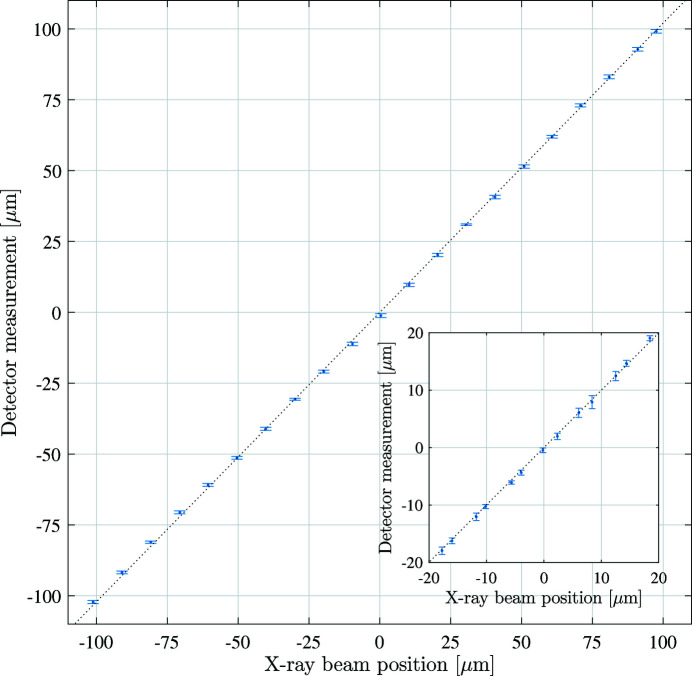
The detector is scanned across the incident X-ray beam in steps of 10 µm. At each point in the scan images are acquired at 100 FPS. The X-ray beam position is obtained using a 2D Gaussian fit of the resulting image. Inset: scan with 3 µm requested step size. The error bars are the standard deviation of the beam position obtained from sequential images acquired at each step.

Fig. 14 ▸ presents the results of these scans. The error bars are the standard deviation in fitted beam position from the sequential detector images acquired at each scan position. They represent the uncertainty on the acquired beam position for the 180 µm FWHM X-ray beam, and are not the intrinsic resolution (point spread function) of the detector. They provide an upper bound on the beam position resolution. The mean standard deviation across the whole scan was 600 nm, or 0.3% of the vertical beam size (FWHM).

## 8. Conclusions   

The pixel detector presented in this paper is capable of transmissively imaging a synchrotron X-ray beam profile. A modulation lock-in readout technique has been demonstrated, enabling all pixels to be read simultaneously, and beam profile images to be obtained at 100 FPS with a 10 ms acquisition period.

Beam motions much smaller than the ‘pixel size’ can be easily resolved by applying 2D Gaussian fitting to determine the centroid, and the resolution of the position measurement is 600 nm for 10 ms acquisitions and 180 µm beam size FWHM. The pixel size of future detectors could easily be varied by changing the spacing between the laser-written electrodes during the fabrication process.

The X-ray absorption of the single-crystal diamond plate is an order of magnitude less than an equivalent fluorescent screen, and produces significantly less X-ray scatter, allowing for permanent installation. It includes no metallization in the beam path and introduces fewer absorption edges that may interfere with beamline experiments. The size of the pixel sensor is limited by the size of available high-quality single-crystal diamond, and is significantly smaller than the range of fluorescent screens that are commercially available. However, this limitation is offset by the advantage of a permanently installed monitor, providing online profile measurements. This possibility is beneficial to many beamlines, allowing real-time monitoring of both the beam position and size. Keeping the transmissive region of the sensor free from metallization also reduces the danger of electrodes being damaged by high incident flux. As a result, this detector could be useful in white-beam applications.

Future work will concentrate on determining the point spread function of the detector by repeating these measurements using a much smaller X-ray beam size, ≤1 µm. The ultimate bandwidth of the detector is yet to be determined. A frequency response analyser will be used to probe any intrinsic bandwidth limitations of the device, and to help determine the maximum achievable frame rate. The conductivity of the graphitic tracks will be more closely examined in future experiments, and higher flux experiments will help to determine if there are any fundamental limitations with the technique arising from large (∼ 100 µA) current flows through the tracks.
